# HomPPI: a class of sequence homology based protein-protein interface prediction methods

**DOI:** 10.1186/1471-2105-12-244

**Published:** 2011-06-17

**Authors:** Li C Xue, Drena Dobbs, Vasant Honavar

**Affiliations:** 1 Department of Computer Science, Iowa State University, Ames, IA 50011, USA; 2 Department of Genetics, Development and Cell Biology, Iowa State University, Ames, IA 50011, USA; 3 Bioinformatics and Computational Biology Program, Iowa State University, Ames, IA 50011, USA

## Abstract

**Background:**

Although homology-based methods are among the most widely used methods for predicting the structure and function of proteins, the question as to whether interface sequence conservation can be effectively exploited in predicting protein-protein interfaces has been a subject of debate.

**Results:**

We studied more than 300,000 pair-wise alignments of protein sequences from structurally characterized protein complexes, including both obligate and transient complexes. We identified sequence similarity criteria required for accurate homology-based inference of interface residues in a query protein sequence.

Based on these analyses, we developed HomPPI, a class of sequence homology-based methods for predicting protein-protein interface residues. We present two variants of HomPPI: (i) NPS-HomPPI (Non partner-specific HomPPI), which can be used to predict interface residues of a query protein in the absence of knowledge of the interaction partner; and (ii) PS-HomPPI (Partner-specific HomPPI), which can be used to predict the interface residues of a query protein with a specific target protein.

Our experiments on a benchmark dataset of obligate homodimeric complexes show that NPS-HomPPI can reliably predict protein-protein interface residues in a given protein, with an average correlation coefficient (CC) of 0.76, sensitivity of 0.83, and specificity of 0.78, when sequence homologs of the query protein can be reliably identified. NPS-HomPPI also reliably predicts the interface residues of intrinsically disordered proteins. Our experiments suggest that NPS-HomPPI is competitive with several state-of-the-art interface prediction servers including those that exploit the structure of the query proteins. The partner-specific classifier, PS-HomPPI can, on a large dataset of transient complexes, predict the interface residues of a query protein with a specific target, with a CC of 0.65, sensitivity of 0.69, and specificity of 0.70, when homologs of both the query and the target can be reliably identified. The HomPPI web server is available at http://homppi.cs.iastate.edu/.

**Conclusions:**

Sequence homology-based methods offer a class of computationally efficient and reliable approaches for predicting the protein-protein interface residues that participate in either obligate or transient interactions. For query proteins involved in transient interactions, the reliability of interface residue prediction can be improved by exploiting knowledge of putative interaction partners.

## Background

Protein-protein interactions are central to protein function; they constitute the physical basis for formation of complexes and pathways that carry out virtually all major cellular processes. These interactions can be relatively permanent or "obligate" (e.g., in subunits of an RNA polymerase complex) or "transient" (e.g., kinase-substrate interactions in a signalling network). Both the distortion of protein interfaces in obligate complexes and aberrant recognition in transient complexes can lead to disease [1].

With the increasing availability of high throughput experimental data, two related problems have come to the forefront of research on protein interactions: i) prediction of protein-protein interaction partners; and ii) prediction of protein binding sites or protein-protein interfaces (PPIs). Although most effort to date has focused on one or the other of these problems, it is possible to use information from predicted protein-protein interaction networks as input for interface prediction methods, and predicted interface residues can be used as input for interaction partner predictions, a concept explored in a recent study of Yip et al. [2]. In the current study, we focus on the prediction of protein-protein interfaces, specifically, the use of sequence homology-based methods to predict which residues of a query protein participate in its physical interaction with a partner protein or proteins.

### Computational Prediction of Protein-Protein Interfaces

Several different genetic, biochemical, and biophysical methods have been used to identify and characterize protein interfaces [1]. These experiments are very valuable and have contributed greatly to our knowledge of protein-protein interfaces. However, the high cost in time and resources required for these experiments call for reliable computational approaches to identify interface residues. In addition to providing important clues to biological function of novel proteins, computational predictions can reduce the searching space required for docking two polypeptides [3].

To distinguish interface residues from non-interface surface residues, a wide range of sequence, physicochemical and structural features have been investigated [3-18], and many in silico approaches to protein-protein interface prediction have been explored in the literature (reviewed in [19-21]). Protein-protein interface prediction algorithms can be classified into three categories: (i) sequence-based methods, which use only the primary amino acid sequence of the query protein as input [3,22-28]; (ii) structure-based methods, which make use of information derived from the structure of the query protein [5,18,29-31]; and (iii) methods that use both sequence and structure derived information in making predictions [32,33].

Several sequence-based protein-protein interface prediction methods have been explored in the literature [3,22-28]. Most, if not all, of these methods, extract for each residue in the query protein, a fixed length window that includes the target residue and a fixed number of its sequence neighbours. Each residue is classified as an interface residue or a non-interface residue based on features of the amino acids in the corresponding window. Various methods differ both in the specific machine learning algorithms or statistical methods employed and in terms of the specific features of the amino acids used. Commonly used features include the identity of the amino acids in the window [27], the amino acid composition of interfaces [34], the physicochemical properties of the amino acids [35], and the degree of conservation of the amino acids (obtained by aligning the query sequence with homologous sequences) [3]. Some studies report substantial improvements in interface residue prediction when predicted structural properties, e.g., solvent surface accessibility and secondary structure of the residues are utilized [21].

A number of structure-based methods [5,18,29-31] or hybrid methods that combine both sequence and structure-derived information [32,33] have been proposed for predicting protein interfaces. The performance of the best-performing sequence-based methods is generally lower than that of structure-based methods (see [21] for a comparison). A possible explanation for the difference in the performance of sequence-based and structure-based protein interface residue predictors is that the latter can trivially eliminate non-surface residues from the set of candidate interface residues and potentially exploit a rich set of features derived from the 3D structures.

The use of structure-based methods, however, is limited to proteins for which the structure of the query protein is available, and the number of solved structures significantly lags behind the number of protein sequences [35]. Even when the structure of a query protein is available, the application of structure-based prediction methods is complicated by conformational changes that take place when some proteins bind to their partners. Structure-based methods rely on structural features extracted from the structure in the unbound state or from a bound complex that has been separated into constituent proteins. It is unclear whether such structural features are indeed reliable predictors of interfaces for proteins that undergo significant conformational changes upon binding [20,36]. Moreover, higher organisms have a large number of intrinsically disordered proteins/regions (IDPs/IDRs) that undergo induced folding only after binding to their partners [37]. Such disordered regions - for which experimental structure information is, by definition, lacking - participate in many important cellular recognition events, and are believed to contribute to the ability of some hub proteins to interact with multiple partners in protein-protein interaction networks [38]. Hence, there is an urgent need for sequence-based methods for reliable prediction of protein-protein interfaces.

### Analysis of Interface Residue Conservation

The relationship between sequence conservation and various aspects of protein structure, interaction, expression, and function has been the focus of many studies over the past decades [39,40], and sequence homology-based methods have been used for predicting both protein structure and protein function [41-53]. Thus, it is natural to ask whether protein-protein interface residues can be reliably identified using sequence homology-based methods. Published studies disagree on whether protein-protein interfaces are more conserved than the rest of the protein sequences. Grishin and Phillips [54], after examining five enzyme families, concluded that the degree of conservation of interfaces is same as that of protein sequences as a whole. The studies by Caffrey et al. [55] as well as Reddy and Kaznessis [56], found that the interacting surface-patches are not significantly more conserved than other surface-patches. Caffrey et al. [55], based on their study of 64 protein-protein interacting chains, found that interface residues are slightly more conserved than the rest of the protein surface residues. Reddy and Kaznessis [56], based on their study of 28 hetero transient and non-transient complexes, found that the fraction of highly conserved interface residues is greater than that of highly conserved non-interface surface residues. They suggested that the number of conserved residue positions is more predictive of protein-protein binding sites than the average conservation index of residues in the target patch. Choi et al. [57] analyzed 2,646 protein interfaces based on a conservation score that measures the position-specific evolutionary rate estimated using a phylogenetic tree [58], and concluded that protein interface residues are more conserved than non-interface surface residues.

Despite the disagreement regarding whether interface residues are conserved or not, several researchers have used conservation of residues to predict protein-protein interfaces with varying degrees of success. For example, the Evolutionary Trace (ET) method [59,60] and its variants [58,61-64] calculate conservation score for each residue using a phylogenetic tree built from a multiple sequence alignment. Residues with conservation scores above a certain threshold are mapped onto the 3D structure of the protein to identify putative binding sites. Carl et al. [65] used a dataset of sixteen transient protein chains to explore the feasibility of predicting protein-protein binding sites based on their membership in structurally conserved surface patches (where conserved patches are identified using structural alignment of a query protein with one or more of its structural homologs). Bordner and Abagyan [66] and Wang et al. [67] calculated evolution rate for each amino acid of protein sequences using phylogenetic trees, and used evolution rate as an attribute along with other physicochemical and sequential attributes to train a SVM classifier for interface residue prediction. Panchenko et al. [68] predicted functional sites of proteins using spatial averages of sequence conservation scores. Shoemaker et al. [69] have recently developed a web server for predicting protein binding sites by inspecting homologous proteins with similar structures. Based on a statistical analysis of target-template sequence alignments on a benchmark dataset of 329 two-chain complexes, Kundrotas and Vakser [70] have shown that it is possible to obtain high quality alignment of interface residues even when the overall alignment quality is rather poor. Specifically, they concluded that in approximately 50% of the complexes considered, the overall accuracy of the modelled interfaces was good enough for guiding docking.

### Overview of the Paper

Against this background, we study a class of sequence homology-based methods for protein-protein interface prediction. We introduce a novel measure of interface conservation that captures the degree to which interface residues in each protein are conserved among its sequence homologs. First, we describe the results of our analysis of the interface conservation among homologous sequences using several large non-redundant datasets of protein-protein interfaces extracted from the Protein Data Bank (PDB) [71], including datasets that allow us to compare "obligate" versus "transient" interfaces. To explore the extent to which interface conservation can be exploited in the prediction of interface residues, we systematically examined the relationship between interface conservation and six sequence-based variables. In one set of experiments, we examined binding interfaces in homologous proteins *without *specifying a specific interaction partner (i.e., non-partner specific, NPS-interfaces). The results of this analysis indicated that interfaces in obligate complexes are, in general, more highly conserved than those in transient complexes. In a complementary set of experiments, we examined interfaces in complexes between specific pairs of proteins (i.e., partner-specific, PS-interfaces). In contrast to the results for NPS-interfaces, by focusing on the interface of each query protein with a *specific *binding partner, we discovered a high degree of sequence conservation in transient PS-interfaces. This analysis revealed that transient interfaces tend to be highly partner-specific.

Second, based on the results of protein interface conservation analysis we propose HomPPI, a class of sequence homology-based approaches to protein interface prediction. We present two variants of HomPPI: (i) NPS-HomPPI (non partner-specific HomPPI), which can be used to predict interface residues of a query protein in the absence of knowledge of the interaction partner; and (ii) PS-HomPPI (partner-specific HomPPI), which can be used to predict the interface residues of a query protein with a specific target protein. The performance of both HomPPI methods was evaluated on several benchmark datasets, including a large non-redundant set of transient complexes. Due to the increasing importance of intrinsically disordered proteins in understanding molecular recognition mechanics and in rational drug design and discovery [72-75], we also tested NPS-HomPPI on two datasets of intrinsically disordered proteins.

Finally, we compare the performance of HomPPI with that of other web-based servers for interface residue prediction, using several performance measures that assess the reliability of correctly predicting, on average, interface and non-interface residues in a given protein. We discuss the relative advantages and limitations of homology-based methods for interface residue prediction.

## Results

To define conditions under which it should be possible to infer protein-protein interface (PPI) residues using conservation of interfaces in homologous proteins and/or complexes, we systematically examined the relationship between interface residue conservation and sequence similarity (based on BLAST alignments). Our analyses are based on the following datasets: Nr6505 (a large non-redundant dataset of protein chains extracted from PDB [71]), Oblig94 and Trans135 (a non-redundant obligate/transient binding dataset taken from [76]), and nr_pdbaa_s2c (BLAST database) (see ***Methods ***for additional details).

### Conservation of PPIs in Non-Partner Specific (NPS) Interfaces

First, we examined the conservation of PPI residues in the absence of knowledge of interaction partners. For this study, we analyzed interfaces in putative homologs (hereafter, we refer to putative homologs as "homologs" for simplicity) of each protein in a large non-redundant dataset, Nr6505. After removing chains with interfaces containing fewer than 3 amino acids, we were left with 5853 chains. For each of the 5853 remaining proteins, we extracted homologs from the nr_pdbaa_s2c database using BLASTP [77] with expectation value (*EVal*) ≤ 10 from the resulting set of homologs, we eliminated those that were nearly identical to the query sequence (to ensure an accurate estimate of conservation). To ensure that the interface residues of the homologs could be reliably determined, we retained only those homologs that were part of complexes with resolution 3.5 Å or better. For each query-homolog pair in sequence alignments generated by BLASTP, we used the interface residues of the homolog(s) to predict the interface residues of the query protein. We calculated the correlation coefficient (CC) between the predicted and actual interface residues of the query protein, and refer to this value as the interface conservation (*IC*) score, i.e., the degree of conservation of interface residues between the query protein and its homologs (see ***Methods ***for details).

We examined the dependence of the interface conservation score on six NCBI BLAST alignment statistics: Expectation value (*EVal*), *Identity Score*, *Positive Score*, *Local Alignment Length (LAL) *and two Alignment Length Fractions (*LAL/Query Length*) and (*LAL/Homolog Length*). The *EVal *is a statistic that estimates the number of hits expected by chance when searching database of a particular size; the lower the *EVal *value, the more significant the score. The *Identity Score *is a measure of the degree of sequence identity between two amino acid sequences. The *Positive Score *returned by BLASTP is the number of positive-scoring matches in an alignment. It takes into account observed substitutions that preserve the physicochemical properties of the original residue. The *LAL *is the length of the local alignment; Alignment Length Fractions are *LAL *normalized by the length of the query or the length of the identified homologous sequence. We represent each query-homolog pair as a six dimensional vector defined by these six variables.

#### Principal Components Analysis of NPS-interface Conservation Space

As a first exploratory step, PCA (Principal Component Analysis) was applied to visualize the relationships between the interface conservation (*IC*) scores and the six BLAST alignment statistics. PCA, which is a dimensionality reduction technique, is typically used to represent dimensions that explain maximum variability and provide a simple and parsimonious description of the covariance structure [78].

**Figure 1:**
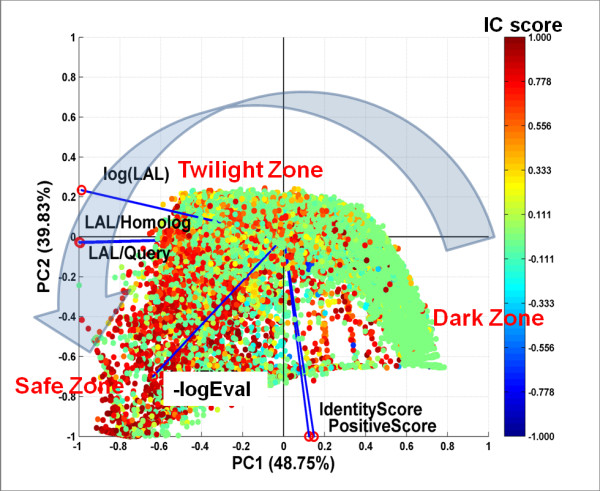
**Principal Component Analysis of Interface Conservation Scores and Sequence Alignment Statistics**. Proteins in the Nr6505 and their homologs were analyzed. The data points in the biplot correspond to the projection of a 6-dimensional vector representing each protein-homolog onto a 2-dimensional space defined by the first and second principal components (PC1 and PC2). Blue lines with red circles at their tips represent the axes of the original 6-dimensional space for the 6 variables used in PCA analysis*: -log(EVal)*, *Identity Score*, *Positive Score*, *log(LAL)*, alignment length fractions (*LAL/query length*) and (*LAL/homolog length*). Each data point is colored according to its computed interface conservation (*IC*) score, with higher *IC *scores (red/orange) indicating higher interface conservation and lower *IC *scores (blue/green) indicating lower interface conservation (see text for details). The large gray arrow indicates the direction of increasing degree of interface conservation, from Dark to Twilight to Safe Zone.

Figure 1 shows a PCA biplot in which each data point, representing a query-homolog pair, is projected from the original 6-dimensional space to a 2-dimensional space defined by the first and second principal components (PC1 and PC2). A large fraction (88.58%) of the variance is explained by the first two principal components (48.75% + 39.83%). Based on *IC *scores, the PCA biplot can be subdivided into three regions that correspond to: (i) Dark Zone: containing query-homolog pairs with poorly conserved interface residues (blue and green data points), corresponding to low values of the CC between predicted and actual interfaces and thus low *IC *scores; (ii) Twilight Zone: containing pairs with moderately conserved interfaces (yellow and orange data points); and (iii) Safe Zone: containing pairs with highly conserved interfaces (red data points).

The PCA analysis allows us to identify highly correlated explanatory variables. In Figure 1, the axes of the original 6 dimensional space are represented as blue vectors with red circles at their tips in the 2-dimensional space defined by PC1 and PC2. Highly correlated vectors (variables) have small angles between them. This type of analysis reveals, for example, that the two Alignment Length Fractions are highly correlated with each other, as are the *Positive Score *and *Identity Score*. Explanatory variables that are highly correlated with each other make similar contributions to the *IC *score.

#### BLAST EVal is a strong indicator of NPS-interface conservation

**Figure 2:**
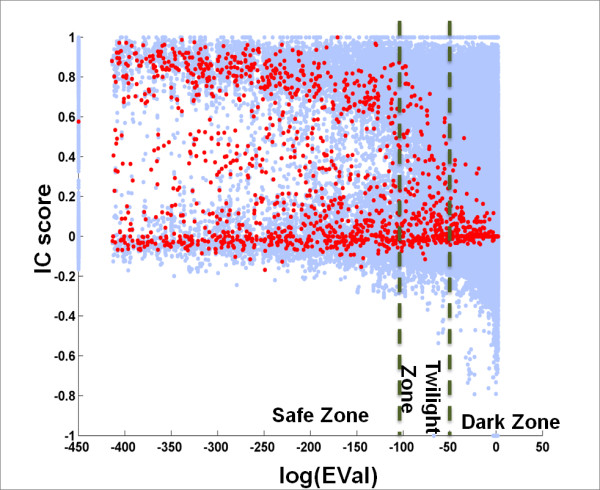
**EVal is a Good Indicator of Interface Conservation**. Each blue dot in the scatter plot corresponds to a query-homolog pair. Red dots are the median values of *IC *scores for a specific *EVal*. To avoid *log(0)*, we set *log(EVal) *= -450 when *EVal = 0*.

We studied the relationship of each individual variable with interface conservation. A scatter plot in which the *IC *score for each query-homolog pair is plotted against *log(EVal) *is shown in Figure 2. One can see that *log(EVal) *is a good indicator of protein interface conservation. When *log(EVal) *> -50 the median values of *IC *scores cluster around 0 (low conservation). In the region of *log(EVal) *≤-50 (that is, *EVal *≤ 1.9287E-022) the medians of *IC *scores increase as the *log (EVal) *decreases. When *log(EVal) *< -100 the medians of *IC *scores tend to be greater than 0.5 (strong conservation).

#### NPS-interface conservation in Twilight/Safe Zone is strongly positively correlated with log(LAL)

**Figure 3:**
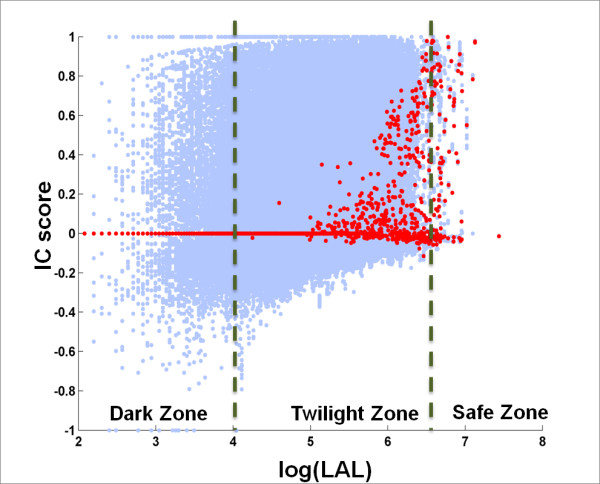
**Interface Conservation (*IC*) Scores are Linearly Related to the Log of the Local Alignment Score (LAL)**. Each blue dot in the scatter plot corresponds to a query-homolog pair. Red dots are the median values of *IC *scores for a specific *LAL*. Note the trend of increasing median *IC *score with *log(LAL) *observed with the transitions from Dark to Twilight to Safe Zone.

Figure 3 is a scatter plot showing the *IC *score for each query-homolog pair plotted against the log of its *LAL *value. We can clearly see that when *log(LAL) *is larger than 4, the medians of *IC *score show a strong positive correlation with *log(LAL)*. When the *LAL *is shorter than 55 residues (*log(LAL) <4*), the probability that interface is conserved in these homologs is low (the medians of the *IC *scores are ~0). We define this region as the Dark Zone. When the *LAL *is longer than 700 residues (*log(LAL)>6.55*), interface conservation is high (the medians of *IC *scores are usually > 0.7). We define this region as the Safe Zone.

#### A high BLAST Positive Score reflects NPS-interface conservation

**Figure 4:**
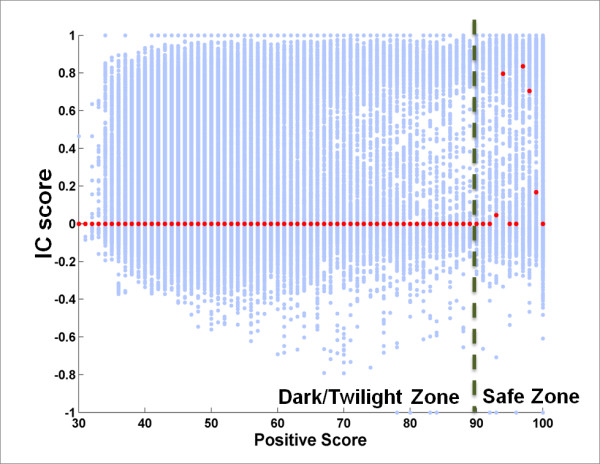
**A High BLAST *Positive Score *Reflects NPS-Interface Conservation**. Each blue dot in the scatter plot corresponds to a query-homolog pair. Red dots are the median values of *IC *scores for a specific *Positive Score*. Note that medians of *IC *scores are near zero until *Positive Scores *become larger than 90%.

The relationship between *IC *scores and the *Positive Scores *of query-homolog alignments is shown in Figure 4. The median values of the *IC *scores begin to increase at a BLAST *Positive Score *of ~ 90%.

We also studied the relationship of *IC *score with the *Identity Score*, and the Local Alignment Length Fractions (*LAL/Query Length*) and (*LAL/Homolog Length*). As expected, the *Identity Score *results were similar to those for the *Positive Score*. The *IC *score was not as strongly linearly related to *LAL *fraction as it was to the *log(LAL) *(data not shown). Taken together, these results provide guidelines for choosing sequence similarity thresholds that reflect the degree of conservation in NPS interfaces.

### NPS-Interface Conservation in Transient versus Obligate Binding Proteins

In light of reports that protein interfaces in transient complexes are not as conserved as those in obligate (permanent) complexes [57], it is interesting to ask whether the query-homolog pairs with near-zero *IC *scores (Figure 2 and Figure 3) tend to involve proteins that participate in transient interactions. To address this question, we further studied the differences in protein interface conservation among proteins that participate in transient versus obligate interactions.

To compare protein interfaces in transient and obligate complexes, we used the Trans135 and Oblig94 dataset obtained from [76], which includes a total of 270 chains from transient and 188 chains from obligate complexes. We extracted the homologs of each chain from nr_pdbaa_s2c using BLASTP with *EVal *≤ 10 Query and homolog proteins with interfaces containing fewer than 3 amino acids were removed, as were homologs that were nearly identical to the query proteins. We extracted 43,115 query-homolog pairs containing chains that participate in transient interactions and 24,212 pairs containing chains that participate in obligate interactions.

**Figure 5:**
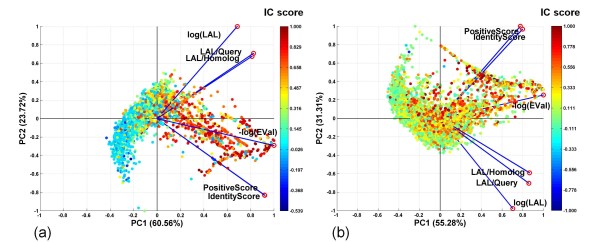
**Principal Component Analysis of Interface Conservation Scores and Sequence Alignment Statistics for Obligate versus Transient Complexes**. The PCA biplots shown are for (a) proteins from obligate complexes and (b) proteins from transient complexes. Please see Figure 1 legend for additional details.

In agreement with previous studies [57], our analyses showed that PPIs are conserved in both obligate and transient binding proteins. As before, we performed PCA to examine the conservation of interfaces as a function of *log(EVal)*, *Identity Score*, *Positive Score*, *log(LAL)*, and alignment length fractions (*LAL/Query Length*) and (*LAL/Homolog Length*). The PCA biplots in Figure 5 show that data points corresponding to different *IC *scores (different colors) are partially segregated, indicating that the six alignment statistics can distinguish query-homolog pairs with highly conserved interface residues (red) from those in which interface residues are not conserved (blue or green).

**Figure 6:**
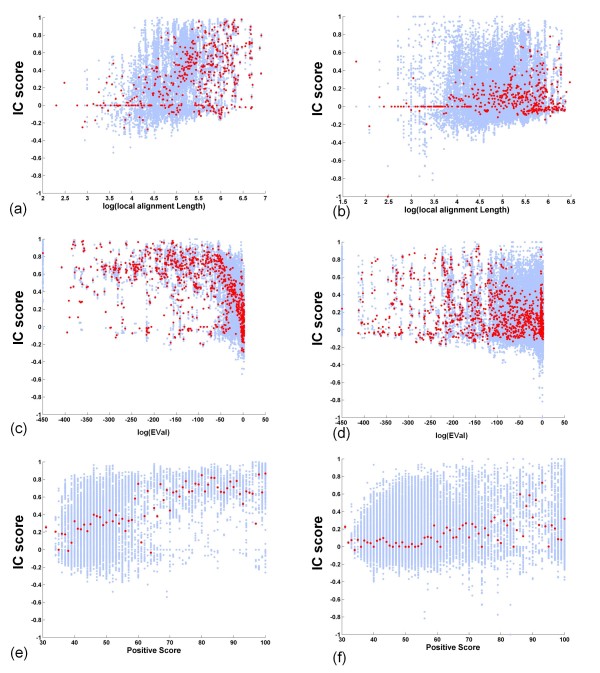
**Comparison of Interface Conservation in Proteins from Obligate versus Transient Complexes**. Proteins from obligate complexes are analyzed in a, c and e (left panels); proteins from transient complexes are analyzed in b, d, and e (right panels). Scatter plots show *IC *scores plotted as a function of: (a, b) *log (local alignment length)*; (c, d) *log (EVal)*; and (e, f) *Positive Score*. Red dots are median values of *IC *scores for a specific value on the x-axis.

The results in Figure 5 also reveal that interface residues in proteins from obligate complexes (left panel) are more conserved among their sequence homologs than those from transient complexes (right panel). Figure 6 further illustrates differences in interface conservation in obligate (left) versus transient complexes (right). The median values of *IC *scores plotted as a function of *log (LAL) *are more frequently above 0 for pairs that involve obligate binding proteins (Figure 6a) than for those that involve transient binding proteins (Figure 6b). Regression analysis of these data confirms that *log(LAL) *for the obligate dataset has a larger coefficient (0.095) than that for the transient dataset (0.052), which confirms that protein interfaces are more conserved in the obligate complexes than in transient complexes analyzed in this study.

Figure 6c reveals an obvious pattern of interface conservation in obligate binding proteins: a strong trend of increasing median *IC *score with decreasing *log(EVal)*. In contrast, Figure 6d shows that for transient binding proteins, more of the median values of *IC *scores cluster around 0, indicating that *log(EVal) *has little relation to interface conservation in transient complexes.

Also, comparison of Figure 6e and 6f reveals that the *Positive Score *is a good indicator of interface conservation in the case of proteins from obligate complexes; however, this is not the case for proteins from transient complexes. For obligate binding proteins, when the *Positive Score *exceeds 45%, the medians of *IC *scores begin to show an increasing trend (Figure 6e). In contrast, in the case of transient binding proteins, medians of *IC *scores do not begin to increase until the *Positive Score *approaches 70% (Figure 6f).

It is important to emphasize that all of the interfaces analyzed above are what we refer to as "non partner-specific" (NPS). That is, the interface residues of a query protein represent the complete set of its interface residues with *all *of its partners. However, a given query protein can interact with different binding partners through different interfaces. A possible explanation for the low *IC *scores for NPS-transient interfaces is that the *union *of all interface residues of a transient binding protein are not highly conserved across its homologs. This does not preclude the possibility that such interfaces are conserved in the context of partner-specific interactions. We investigate this possibility in the following section.

### Conservation of PPIs in Partner-Specific (PS) Interfaces

To examine the conservation of partner-specific (PS) interfaces in transient protein complexes, we again used the Trans135 dataset of protein pairs that participate in transient interactions [76]. For each of the proteins in an interacting pair, we separately extracted the corresponding homologs, using BLASTP with expectation value *EVal≤10 *against the nr_pdbaa_s2c database. We removed homologs that are part of complexes with resolution worse than 3.5 Å. If query proteins A and B form a complex A-B, and have homologs A' and B' that interact in a complex A'-B', we consider A'-B' as a ***homo-interolog ***of A-B. To ensure an accurate estimate of conservation, from the resulting set of homo-interologs, we eliminated those that were within the same PDB complex as the query proteins, and those that were nearly identical to the query pairs (see ***Methods ***for additional details). For each protein chain in a query pair, we use the interface residues of its homolog in a homo-interolog to infer the PS interface residues of the query protein chain. Thus, we use the interface residues of A' in the homo-interolog (A'-B') of query pair A-B to infer the interfaces of A with B, based on the sequence alignment between A and A' obtained using BLASTP. We measure the similarity between a pair of interacting proteins A-B and its homo-interolog A'-B', in terms of the metrics for the quality of sequence alignment between A and A' and between B and B', using the six BLAST alignment statistics described above.

We used PCA of 3, 456 candidate homo-interologs to explore the relationship between interface conservation (*IC *score) and the six alignment statistics computed from the predicted PS interfaces, e.g., of chain A when it interacts with B, using known interfaces of A' with B'. This analysis revealed that much of the observed variance in *IC *scores is explained by three factors: (i) the average *log (EVal)*; (ii) the average *Positive Score *of the homo-interolog and (iii) the alignment fractions *Frac_A_*, *Frac_A_*_'_, *Frac_B_*, and *Frac_B_*_' _computed from the alignments of constituent chains (A with A' and B with B') (see ***Methods ***for additional details).

**Figure 7:**
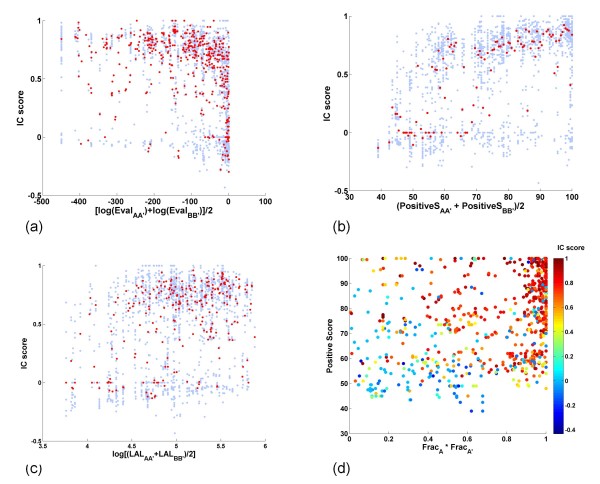
**PS-Interface Conservation in Transient Complexes**. Homo-interologs corresponding to complexes in the Trans135 dataset were analyzed (see text for details). (a-c) Scatter plots show *IC *scores (blue dots) plotted as a function of: (a) *log EVal*; (b) *Positive Score*; (c) *log LAL*. Red dots are median values of *IC *scores for a specific value on the x-axis. (d) Scatter plot of *Positive Score *as a function of *Frac_A _*× *Frac_A_*_'_. Each data point in (Fig. 7d only) is colored using according to its *IC *score.

The results in Figure 7 show that transient interfaces are highly conserved in homo-interologs. The trend of increasing median *IC *scores, as a function of decreasing *logEval *(Figure 7a) or increasing *Positive Score *(Figure 7b) or the combination of *Positive Score *and *Frac_A _*× *Frac_A_*_' _is clear (Figure 7d). The trend of increasing *IC *scores as a function of *Frac_B _*× *Frac_B_*_' _is similar to that as a function of *Frac_A _*× *Frac_A_*_' _(data not shown). In contrast, the, *logLAL*, which is the average of alignment length between A and A', and between B and B', is not strongly correlated with interface conservation for PS-interfaces (Figure 7c).

A comparison of the results for PS-interface conservation in transient complexes here (Figure 7a and 7b) with those obtained for NPS-interface conservation in transient complexes above (Figure 6d and 6f), reveals that the conservation of transient interfaces can be detected easily when the binding partner sequence information is utilized. The seemingly weak conservation of interfaces in transient complexes shown in Figure 6 is thus a consequence of the *specificity *of transient interfaces for different partners. Therefore, we conclude that interfaces in transient complexes are both highly partner-specific and highly conserved, when their partner-specificity is taken into account.

### HomPPI - Homologous Sequence-Based Protein-Protein Interface Prediction

Based on the results of our analysis of protein interface conservation described above, we developed HomPPI, a family of sequence homology-based algorithms for protein interface prediction. We implemented two variants of HomPPI:

1. **NPS-HomPPI **- Given a query protein sequence, NPS-HomPPI searches the nr_pdbaa_s2c database to identify homologous proteins that are components of experimentally determined complexes with one or more other proteins. NPS-HomPPI labels a residue of the query sequence as an "interface" residue if a majority of residues in a selected subset of homologs in alignment of the query sequence with its homologs are interface residues, and as "non-interface" residue otherwise. Specifically, given a query protein, we first use NPS-HomPPI to search for sequence homologs within the Safe Zone. If at least one homolog in the Safe Zone is found, NPS-HomPPI uses the Safe homolog(s) to infer the interfaces of the query protein. Otherwise, the process is repeated to search for homologs in the Twilight Zone or the Dark Zone. If no homologs of the query protein can be identified in any of the three zones, NPS-HomPPI does not provide any predictions. The Safe, Twilight, and Dark Zone homologs of the query protein sequence to be used for interface prediction are identified by searching the nr_pdb_s2c database using BLASTP with thresholds based on the interface conservation analysis (see ***Methods ***Section for details) (after removing the query sequence and any highly similar sequences from the same species as the query sequence, in order to allow unbiased evaluation of the performance of NPS-HomPPI).

2. PS-HomPPI - Given the sequences of a query protein A and its putative binding partner B, PS-HomPPI searches the nr_pdbaa_s2c database to identify homologous *complexes *i.e., the homo-interologs of A-B. PS-HomPPI labels a residue of the query sequence as an "interface" residue (with respect to its putative binding partner) if a majority of the residues in the corresponding position in homologous complexes are interface residues, and as "non-interface" residues otherwise. PS-HomPPI uses homo-interologs in Safe and Twilight Zones to make predictions. The PS-HomPPI prediction process is thus analogous to that for NPS-HomPPI, using thresholds for "close homo-interologs" based on the results of interface conservation analysis of PS-interface conservation (see ***Methods ***Section for additional details).

### Performance Evaluation of HomPPI Methods

We report several performance measures that provide estimates of the reliability of interface (and non-interface) residue predictions obtained using the HomPPI family of predictors. We compare the performance of HomPPI predictors with several state-of-the-art interface prediction methods on a benchmark dataset. We evaluate the effectiveness of HomPPI in predicting the interface residues of disordered proteins. Finally, we compare the partner-specific and non-partner-specific versions of HomPPI.

We focus our discussion on results using several performance measures that assess the effectiveness of the methods in reliably predicting, on average, the interface and non-interface residues of any given *protein *(See ***Methods ***for details). However, because several of the published studies report performance measures that assess the effectiveness of the methods in reliably assigning interface versus non-interface labels, on average, to any given protein *residue*, we also include results using "residue-based" performance measures in Supplementary Materials (See http://homppi.cs.iastate.edu/supplementaryData.html).

#### (i) NPS-HomPPI Performance on the Benchmark180 Dataset

Among the 180 protein sequences in the Benchmark180 dataset (taken from [79]), 125 sequences had at least one homolog that met the thresholds for the Safe or Twilight Zones, based on zone boundaries determined using Trans135 (Table 1). We examined the performance of NPS-HomPPI in predicting interface residues on each of the four different protein complex types in Benchmark180. As shown in Table 2, NPS-HomPPI performed best on obligate homodimers, in terms of CC (0.76), sensitivity (0.83), specificity (0.78) and accuracy (0.94). Performance on obligate heterodimers was comparable, although slightly lower. NPS-HomPPI performance on transient interfaces was substantially lower than on obligate interfaces. For transient enzyme inhibitor complexes, the accuracy was 0.86, with a CC of 0.53; for transient non enzyme-inhibitor complexes, the accuracy was 0.83, with a CC of 0.45. These results are consistent with the finding from our statistical analyses that NPS-obligate interfaces are more conserved than NPS-transient interfaces in their homologs.

**Table 1:** Boundaries of Safe, Twilight and Dark Zones used by NPS-HomPPI^a^.

	*log(EVal)*	≤ -100
Safe Zone	*Positive Score*	≥80%
	*log(LAL)*	≥5.2
	*log(EVal)*	≤ -50
**Twilight Zone 1^b^**	*Positive Score*	≥65%
	*log(LAL)*	≥4

	*log(EVal)*	≤ 1
**Twilight Zone 2^b^**	*Positive Score*	≥60%
	*log(LAL)*	≥4

	*log(EVal)*	≤ 1
**Dark Zone**	*Positive Score*	≥0
	*log(LAL)*	≥0

^a ^Thresholds were chosen based on interface conservation analysis of proteins in Trans135.

^b ^Twilight Zone is divided into two sub-zones: Twilight Zone 1 with stricter thresholds, and Twilight Zone 2 with looser thresholds.

**Table 2:** Interface Residue Prediction Performance of NPS-HomPPI on Benchmark180.

Binding Type	Homology Zone	Prediction Coverage	CC^P^	Sensitivity^P^	Specificity^P^	Accuracy^P^
Enzyme-inhibitor,- Transient	Safe/Twilight	67% (24/36)	0.53	0.67	0.58	0.86

Non-enzyme-inhibitor-Transient (NEIT)	Safe/Twilight	60% (18/30)	0.45	0.54	0.58	0.83

Hetero-dimer - Obligate	Safe/Twilight	85% (23/27)	0.63	0.72	0.69	0.88

Homo-dimer - Obligate	Safe/Twiight	69% (60/87)	0.76	0.83	0.78	0.94

Prediction coverage reflects that predictions are made only on proteins for which HomPPI can identify Safe/Twilight Zone homologs.

We also evaluated the prediction performance of NPS-HomPPI using homologs with different degrees of sequence homology. In Table 3, the prediction performance is shown separately for sets of test proteins for which HomPPI can identify at least one homolog in Safe, Twilight, or Dark Zones. As expected, Safe Zone homologs consistently gave the most reliable prediction performance for all four types of complexes (CC values ranged from 0.55 to 0.84). Both obligate and transient interfaces were predicted with moderate to high reliability (CC values ranged from 0.12 to 0.67) even using only distant homologs from the Twilight or Dark Zones.

**Table 3:** Prediction Performance of NPS-HomPPI using Homologs from the Safe, Twilight, Dark Zones.

Binding Type	Homology Zone	Prediction Coverage	CC^P^	Sensitivity^P^	Specificity^P^	Accuracy^P^
	Safe	14% (5/36)	0.55	0.62	0.57	0.94
Enzyme-inhibitor, - Transient	Twilight	50% (18/36)	0.52	0.69	0.58	0.83
	Dark	19% (7/36)	0.12	0.23	0.20	0.83
	**Total**	**83% (30/36)**	**0.44**	**0.58**	**0.50**	**0.85**

	Safe	23% (7/30)	0.56	0.64	0.60	0.91
Non-enzyme-inhibitor, - Transient (NEIT)	Twilight	37% (11/30)	0.37	0.48	0.57	0.78
	Dark	33% (10/30)	0.36	0.37	0.50	0.86
	**Total**	**93% (28/30)**	**0.42**	**0.48**	**0.55**	**0.84**

	Safe	52% (14/27)	0.70	0.81	0.72	0.91
Hetero-dimer, - Obligate	Twilight	33% (9/27)	0.52	0.58	0.64	0.82
	Dark	15% (4/27)	0.44	0.66	0.47	0.80
	**Total**	**96% (26/27)**	**0.60**	**0.71**	**0.66**	**0.86**

	Safe	38% (33/87)	0.84	0.90	0.84	0.96
Homo-dimer, - Obligate	Twilight	31% (27/87)	0.67	0.74	0.71	0.91
	Dark	28% (24/87)	0.36	0.47	0.44	0.84
	**Total**	**97% (84/87)**	**0.65**	**0.73**	**0.68**	**0.91**

#### (ii) Comparison of NPS-HomPPI with other PPI Prediction Servers

Direct comparison of NPS-HomPPI with other methods described in the literature is complicated by the limited availability of implementations of the underlying methods (many of which are available only in the form of servers), and differences in the choice of training and evaluation datasets, evaluation procedures and evaluation measures [80]. Hence, we limit our comparisons of HomPPI with five state-of-the-art methods available as web-based servers: Promate [18], Cons-PPISP [33,81], meta-PPISP [82], PIER [83] and PSIVER[22]. All of these methods except PSIVER take advantage of both sequence and experimentally determined protein structure of the query proteins. They have been reported to be among the best performing methods currently available for predicting PPIs (see [20,21] for reviews). PSIVER is one of the most recently published methods for interface residue prediction that only uses protein sequence-derived information. Although direct comparisons of the data representation and the algorithms used by PSIVER with those used by other sequence-based interface residue predictors are currently not available, PSIVER has been reported to outperform two other sequence-based servers: ISIS [3] and the sequence-based variant (made available as an experimental version in 2008) of SPPIDER [84].

Promate samples the protein surface using circular patches around a set of anchoring dots and estimates the probability that each surface dot belongs to an interface, based on the distribution of various physicochemical properties within interface and non-interface patches. Cons-PPISP is a consensus method that combines six neural networks trained on six datasets. Meta-PPISP is a consensus method that combines the output from cons-PPISP, Promate, and PINUP [85]. PIER relies on partial least squares (PLS) regression of surface patch properties of the query protein. PSIVER uses PSSM profiles and predicted solvent accessibility as input features, and uses a Naïve Bayes classifier with parameters obtained using kernel density estimation. Because NPS-HomPPI does not take structural information into account, to compare its performance with the structure-based servers, we mapped the interfaces predicted by each server onto the full sequence of each query protein in order to evaluate prediction performance on the entire protein sequence.

**Figure 8:**
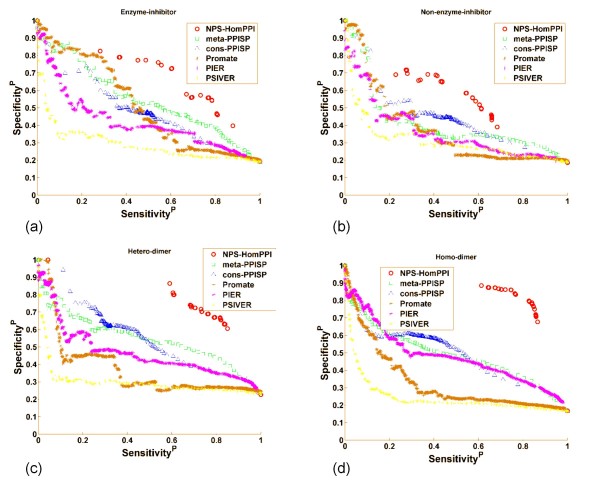
**Performance of NPS-HomPPI Compared with Web-based PPI Servers**. Performance was evaluated on four different protein complex types from Benchmark180: (a) Enzyme-inhibitors, transient. (b) Non-enzyme-inhibitors (NEIT), transient. (c) Hetero-dimers, obligate. (d) Homo-dimers, obligate. Servers compared were: NPS-HomPPI: red circles; Meta-PPISP: green squares; Cons-PPISP: blue triangles; Promate: brown stars; PIER: purple stars; PSIVER: yellow stars.

We compared the performance of NPS-HomPPI with all five PPI servers on a subset of the Benchmark180 dataset [79], specifically, 125 out of 180 proteins for which NPS-HomPPI was able to identify homologs in the Safe or Twilight zones. The sensitivity-specificity plots (also called precision-recall plots) are shown in Figure 8. Each data point corresponds to a different classification threshold value. The prediction score of NPS-HomPPI is simply the normalized vote (for each residue total votes for interfaces from homologs are normalized by the number of homologs) from 10 (or fewer available) homologs. Thus, NPS-HomPPI produces a limited number of distinct prediction scores.

For the two transient complex types, enzyme-inhibitors (Figure 8a) and transient non-enzyme-inhibitors, transient (Figure 8b), NPS-HomPPI consistently outperforms Promate, PIER, meta-PPISP, cons-PPISP, and PSIVER except for sensitivity values lower than 0.2 (which is very low to be useful in practice). On both obligate heterodimers (Figure 8c) and homodimers (Figure 8d), NPS-HomPPI outperforms all five servers across the full range of sensitivity and specificity values for which it can generate homology-based predictions. It should be noted that structure-based methods predict which *surface *residues are interface residues. In contrast, sequence-based methods have the more challenging task of identifying interface residues from the set of *all *residues. In other words, structure-based methods can trivially eliminate all non-surface residues from the set of candidate interface residues. Viewed in this light, the observed predictive performance of NPS-HomPPI, a purely sequence-based method, suggests that it is possible to make reliable non-partner-specific interface residue predictions using only the sequences of a protein by taking advantage of the conservation of interfaces in the context of non-partner-specific interactions.

#### (iii) Performance of NPS-HomPPI on Intrinsically Disordered Proteins

Intrinsically disordered proteins (IDPs) and proteins containing intrinsically disordered regions (IDRs) are attractive targets for drug discovery [73]. The lack of defined tertiary structure in IDPs/IDRs poses a major challenge to structure-based interface prediction methods. Hence, we compared the performance of NPS-HomPPI with ANCHOR [86], a recently published method for the prediction of binding regions in disordered proteins. For this comparison, we used two non-redundant disordered protein datasets, S1 and S2, recently collected by Meszaros et al. [87]. Some of the test proteins are based on data from NMR structures. In order to compare NPS-HomPPI with ANCHOR on the largest possible number of cases available to us, we extracted interface residues from these NMR cases; however, we used only sequence homologs with interface residues determined from X-ray structures to make predictions.

**Figure 9:**
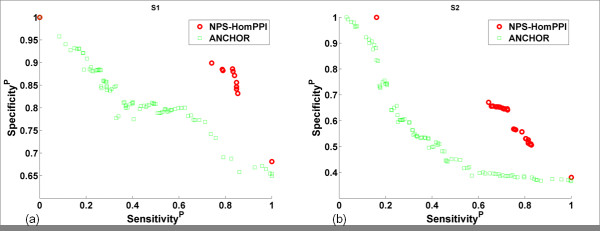
**Performance of NPS-HomPPI Compared with ANCHOR in Predicting Interface Residues in Disordered Proteins**. Two datasets of disordered proteins were used: (a) S1: short disordered proteins. (b) S2: long disordered proteins. NPS-HomPPI: red circles; ANCHOR: green squares.

Figure 9 shows the performance comparison of NPS-HomPPI with ANCHOR on the prediction of interface residues in disordered proteins. NPS-HomPPI significantly outperforms ANCHOR over a broad range of sensitivity and specificity for both short as well as long disordered proteins for which sequence homologs are available in Safe, Twilight or Dark Zones (Figure 9a and 9b respectively). For example, as shown in Figure 9b, on the S2 dataset, at a prediction sensitivity value of 0.70, ANCHOR achieves a specificity of ~0.40, whereas NPS-HomPPI achieves a specificity of ~0.64.

At present, NPS-HomPPI has relatively high prediction coverage for long disordered proteins (78%; 31 out of 40 interfaces of disordered proteins), but lower coverage for short disordered proteins (50%; 28 out of 56 interfaces of disordered proteins). This is in part due to that fact that many disordered proteins available in the PDB have only NMR structures, which were excluded from the current study. Incorporation of data from NMR structures in the future can be expected to increase the coverage of NPS-HomPPI for disordered proteins.

#### (iv) Performance of NPS-HomPPI versus PS-HomPPI

Our analysis of the conservation of PS-transient interfaces described earlier suggests that many interfaces in transient protein complexes are highly partner-specific. Thus, we implemented a variant of HomPPI, designated PS-HomPPI, to evaluate the possibility that prediction of interface residues, especially in transient complexes, can be improved by using sequence information about specific binding partners, when available.

We first evaluated the performance of PS-HomPPI on a transient complex dataset, Trans135 (dimers from the dataset in [76]). PS-HomPPI found at least one homo-interolog that meets the Safe or Twilight similarity thresholds for 60% (162/270) proteins in the Trans135 dataset. Overall, PS-HomPPI had an average CC of 0.65, sensitivity of 0.69, specificity of 0.70 and accuracy of 0.92.

To investigate whether the partner information is, in fact, helpful in predicting interfaces we directly compared the performance of PS-HomPPI with NPS-HomPPI on the Trans135 dataset. In Trans135, there were 139 out of 270 chains that for which predictions could be generated by both NPS-HomPPI (using homologs) and PS-HomPPI (using homo-interologs) from the Safe or Twilight zones (see ***Methods ***for details).

**Figure 10:**
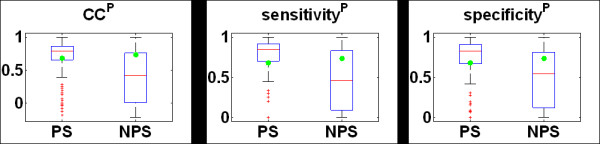
**Performance comparison of PS-HomPPI and NPS-HomPPI**. Only proteins for which predictions could be generated by both PS-HomPPI and NPS-HomPPI (139 out of 270 chains from Trans135) were used in this evaluation. The lower (Q1), middle (Q2) and upper (Q3) quartiles of each box are 25th, 50th and 75th percentile. Interquartile range IQR is Q3-Q1. Any data value that lies more than 1.5 × IQR lower than the first quartile or 1.5 × IQR higher than the third quartile is considered an outlier, which is labelled with a red cross. The whiskers extend to the largest and smallest value that is not an outlier. Averages are marked by green dots.

The results shown in Figure 10 indicate that, at least for transient interfaces in the Trans135 dataset, PS-HomPPI outperforms NPS-HomPPI. Although the average values over proteins (green dots) for CC, sensitivity and specificity are similar, the median values (the red bar in the box) for PS predictions (left panel) are much higher than that for NPS predictions (right panel). Also, the observed variance (length of the box) of PS predictions (left panel) is much smaller than that of NPS predictions (right panel). These results suggest that the reliability of interface residue predictions can be improved by exploiting the knowledge of the binding partner of a query protein.

## Discussion

### Protein Interface Conservation across Structure Space

The study of protein interface conservation among proteins with similar structures has received considerable attention in recent years. By analyzing the structural similarity of representative protein-protein interfaces in dimeric proteins, Gao and Skolnick [88] showed that the vast majority of native interfaces have a close structural neighbor with similar backbone Cα geometry and interface contact pattern.

In a related study, Zhang et al. [89] explored the conservation of interface residues among structural neighbors of a query protein (i.e., proteins that share the same SCOP family, superfamily or fold, or a high degree of structural similarity regardless of their SCOP classification). They showed that: (i) interfaces are indeed conserved among structural neighbors; (ii) the degree of interface conservation is most significant among proteins that have a clear evolutionary relationship. They further showed that conservation of interface residues among structural neighbors can be successfully exploited to predict protein-protein interfaces based on protein structure information.

To investigate the extent to which conservation of interface residues can be used to improve the prediction of protein-protein interfaces based on protein sequence information, we systematically studied interface conservation across *sequence *space. Our results demonstrate that protein interfaces from different binding types are conserved among proteins with homologous sequences. We further showed that the degree of conservation of interfaces is even greater when putative interaction partners are taken into account. The *IC *score, our measure of interface conservation, unlike those used in previous studies [57] (e.g., residue conservation in sequence alignments), makes direct use of experimentally determined interface residues to measure the degree of interface conservation. Specifically, the *IC *score directly measures the extent to which the interface residues of sequence homologs of a query protein are predictive of the interface residues of a query protein. Hence, the *IC *score provides the basis for setting the parameters of our sequence homology-based interface prediction methods.

### Distance Functions for Identifying Putative Homologs with Conserved Interfaces

Because we do not know the *IC *score for a query sequence with unknown interface residues, we identified several statistics associated with the BLASTP alignment of a query sequence with its homologs that are correlated with the *IC *score. We found that interface residues of a query protein can be reliably predicted from the known interfaces of its homologs (and in the case of partner-specific predictions, the homologs of its interaction partner as well) when the homologs are selected taking into account measures of quality of sequence alignment, specifically NCBI BLAST sequence alignment statistics. The HomPPI methods presented here use simple linear combinations of BLAST sequence alignment statistics, determined using PCA analysis of the relationship between the statistics and the *IC *score. It would be interesting to explore optimal, perhaps non-linear, combinations of parameters to maximize the desired performance criteria (e.g., sensitivity, specificity, or some combination thereof).

### Conservation of Interfaces in Obligate and Transient Complexes

Our results are consistent with previous studies [57], in that we found interface residues to be more highly conserved than non-interface residues, in both obligate and transient complexes. We also found that when information regarding the specific binding partner of a query protein is not taken into account in estimating the conservation score, interfaces in transient complexes appear to be less highly conserved than those in obligate complexes. Our results further show that transient interfaces are highly partner-specific, and that the partner-specific interfaces in transient complexes are, in fact, highly conserved. Interfaces of intrinsically disordered proteins that nevertheless form ordered complexes with globular proteins are also highly conserved (see below).

### Interfaces of Disordered Proteins Are Highly Conserved and Non Partner-Specific

Compared with its performance on transient binding proteins in the Benchmark180 dataset, NPS-HomPPI performs much better on interfaces of disordered proteins in the S1 and S2 datasets. This is consistent with the conclusion of Meszaros et al. [90] that interfaces of intrinsically disordered proteins are evolutionarily conserved. The high degree of conservation of interface (binding) regions in IDPs also reflects the important biological functions in which many disordered proteins participate. It is believed that the flexibility of disordered binding regions may facilitate the binding of IDPs using the same set of binding residues to different binding partners (at different times) [91]. Our results suggest that this specialized disorder-to-order transition as a result of binding may be associated with a high degree of interface conservation.

The conservation of interfaces in IDPs may contribute to the generally successful application of interface residue predictors to interfaces in IDPs. Several groups have developed methods for predicting disordered binding regions, including PONDR VL-XT [92,93], ANCHOR [86], and other examples reviewed in [94], that have produced encouraging results. The success of these predictors suggests that at least some sequence features are likely to be conserved within binding regions of different IDPs.

The fact that disordered interfaces can be reliably inferred by NPS-HomPPI indicates that disordered interfaces are non-partner-specific, which is consistent with findings that these proteins are able to bind a broad range of ligands through common binding regions [95,96].

### Performance of HomPPI Compared with Published Methods

Our results show that whenever the interfaces of the close sequence homologs of a query protein are available, NPS-HomPPI outperforms several state-of-the-art protein interface prediction servers (many of which take advantage of the structure of the query protein), over a broad range of sensitivity and specificity values. In the case of transient complexes (Figure 8a and 8b), NPS-HomPPI consistently outperforms Promate, PIER, meta-PPISP, cons-PPISP, and PSIVER except for sensitivity values lower than 0.2. On obligate dimers (Figure 8c and 8d), NPS-HomPPI significantly outperforms all five servers across the full range of sensitivity and specificity values for which it can generate homology-based predictions. These results strongly suggest that it is possible to reliably predict protein interface residues using only sequence information whenever the interface residues of sequence homologs of the query protein are known. Each of the webbased PPI servers with which we compared our NPS-HomPPI server, except PSIVER, take advantage of the structure of the query proteins to determine surface residues, and restrict the predicted interface residues to a subset of the surface residues. This trivially reduces the number of false positive interface residue predictions (relative to the total number of residues in the query protein) which, in turn, yields a substantial increase in the specificity of interface predictions produced by structure-based servers. Consequently, purely sequence-based protein interface prediction servers have a handicap relative to structure-based prediction servers. When viewed in this light, performance of NPS-HomPPI relative to the state-of-the-art protein interface prediction methods is especially impressive.

The HomPPI methods for interface residue prediction do have an important limitation, however, in that they rely on the availability putative homologs for which experimentally-determined structures of bound complexes are available in the PDB. One may ask whether the coverage of the HomPPI family of protein-protein interface prediction methods is broad enough to be sufficiently useful in practice. We address this question below.

### Prediction Coverage of HomPPI Methods

The current coverage of HomPPI protein interface prediction methods can be assessed from our results as follows:

#### NPS-HomPPI

**• Benchmark180 dataset: **NPS-HomPPI found at least one homolog that meets the similarity thresholds for Safe or Twilight Zones for 73% (83/114) of the obligate binding chains (homo and hetero-dimers). Among these, 82% (68/83) were predicted with *both *sensitivity and specificity ≥0.50, simultaneously. Similarly, at least one homolog was found for 62% (42/66) of transient binding chains (enzyme-inhibitors and non-enzyme inhibitors) in this dataset. Among these 55% (23/42) were predicted with *both *sensitivity and specificity ≥0.5.

**• Trans135 dataset**: In the case of transient query proteins in the Trans135 dataset, NPS-HomPPI found at least one homolog that meets the similarity thresholds for Safe or Twilight Zones for 75% (202/270) of chains. Among these, 37% (74/202) were predicted with *both *sensitivity and specificity ≥0.5.

**• Disordered protein datasets S1 and S2**: In the case of disordered proteins, NPS-HomPPI found at least one homolog that meets the similarity thresholds for Safe or Twilight or Dark Zones for 50% (26/52) of interfaces of disordered proteins in S1, the short disordered protein set, and 75% (30/40) of interfaces of disordered proteins in S2.

#### PS-HomPPI

**• Trans135 dataset**: PS-HomPPI found at least one homo-interolog that meets the Safe or Twilight similarity thresholds for 60% (162/270) proteins in the Trans135 dataset. Among these, 80% (130/162) where predicted with sensitivity and specificity ≥0.5, simultaneously.

Based on these results, we estimate that, at present, the coverage of the HomPPI protein interface prediction methods is in the range of 60-70% of all query proteins. As the structural genomics projects currently underway generate increasing numbers of structures of protein-protein complexes [97], we can expect corresponding increases in the coverage of HomPPI family of protein interface prediction methods. In the meantime, one can envision hybrid methods that combine HomPPI with one or more machine learning based methods that do not require the availability of putative homologs for which experimentally determined structures of bound complexes are available in the PDB.

### Parameters for HomPPI Can Be Relaxed for Obligate Interactions

The current default parameters for HomPPI are intentionally rather stringently set based on the results of our statistical analysis of interface conservation using Trans135, which is a dataset of transient binding proteins. Our analyses suggest that NPS-HomPPI has wider Safe and Twilight Zones for obligate binding proteins than for transient binding proteins. Furthermore, even Dark Zone homologs yield interface predictions that are accurate enough to be useful in practice, with average specificity of 0.47 and sensitivity of 0.66 for hetero-obligate dimers, average specificity of 0.44 and sensitivity of 0.47 for homo-obligate dimers (see Table 3). Therefore, for obligate interactions, if a query protein has little sequence similarity with proteins in the PDB, the thresholds of NPS-HomPPI can be relaxed to allow identification of more distant homologs with potentially conserved interfaces that still provide reliable interface predictions.

### Prediction of Binding Partners vs. Prediction of Interface Residues

Protein interface (binding site) predictions and protein interaction (partner) predictions answer closely related, but different questions. Non partner-specific protein ***interface ***predictors are designed to identify the ***residues ***in a query protein that are likely to make contact with the residues of one or more unspecified interaction partner proteins. Partner-specific protein interface predictors are designed to identify the residues in a query protein that are likely to make contact with residues of a putative interaction partner protein. In contrast, protein ***interaction ***predictors are designed to predict whether or not a given pair of proteins is likely to interact [98-101]. Although our study does not directly address the latter question, it is possible to use PS-HomPPI predictions to determine whether or not two query proteins interact: Given a pair of protein sequences, say *A *and *B*, we can first use PS-HomPPI to predict the interface residues of *A *with its putative partner *B*; and the interface residues of *B *with its putative partner *A*. If, in both cases, some number of interface residues are predicted, we can infer that proteins *A *and *B *are likely to interact with each other. Conversely, it is possible to use information from predicted protein-protein *interactions *to refine *interface *predictions. Yip et al. [2] have proposed an approach to utilize residue level information to improve the accuracy of protein level predictions, and *vice versa*. They have shown that a two-level machine learning framework that allows information flow between the two levels through shared features yields predictions that are more accurate than those obtained independently at each of the levels.

### Using Interface Predictions to Steer Docking and to Rank Docked Conformations

Reliable partner-specific interface predictions can be used to restrict the search space for protein-protein docking by specifying the contacts that need to be preserved in the docked conformation. It is also possible to rank the conformations produced by docking, based on the degree of overlap between the interface of a query protein and its binding partner in the docked conformation with the interface generated by a partner-specific interface prediction method, e.g. PS-HomPPI. In related work[102], we have shown that PS-HomPPI provides reliable interface predictions on a large subset of a Docking Benchmark Dataset, and is both fast and robust in the face of conformational changes induced by complex formation. The quality of the ranking of docked conformations by PS-HomPPI interface prediction is consistently superior to that produced using ClusPro cluster-size-based and energy-based criteria for 61 out of 64 docking complexes for which PS-HomPPI produces interface predictions[102].

## Conclusions

We studied a large number of sequence alignments between protein pairs with known interfaces to explore the conditions under which conservation of protein interface residues, as determined by the alignment of a query sequence against its homologs/homo-interologs, can be used to reliably predict protein-protein interfaces. Based on the results of these analyses, we developed HomPPI, a simple sequence-based method for predicting interface residues based on the known interface residues in homologous sequences. HomPPI has two variants: NPS-HomPPI (for predicting interface residues of a query protein with unspecified interaction partners) and PS-HomPPI (for predicting interface residues of query proteins with a specified putative interaction partner).

Our systematic evaluation of NPS-HomPPI showed that, when close homologs can be identified, NPS-HomPPI can reliably predict interface residues in both obligate and transient complexes, with a performance that rivals several state-of-the-art structure-based interface prediction servers. NPS-HomPPI can also be used as a reliable tool for identifying disordered binding regions. In this regard, NPS-HomPPI has an advantage over structure-based interface predictors, which cannot be used to predict binding sites in disordered regions of proteins because they do not form stable structures in their unbound state. In addition, the HomPPI family of interface prediction methods are fast enough for proteome-wide analyses.

Many studies on *in silico *identification of protein interfaces have been published in the past decade. However, despite the fact that many proteins are very specific in their choice of binding partners, the majority of studies focus on only one side of the bound complex. In this study, we implemented a novel partner-specific protein interface prediction method, PS-HomPPI, which infers interface residues based on known interfaces in the homo-interologs, i.e., complexes formed by homologs of the query protein and its putative interaction partner. When homo-interologs can be identified, PS-HomPPI can reliably predict highly partner-specific transient interfaces.

Although our focus in this study was on prediction of protein-protein interfaces, these methods could be useful in other settings, such as sequence-based prediction of protein-DNA, protein-RNA, and protein-ligand interfaces, and the prediction of B and T cell epitopes.

Both NPS-HomPPI and PS-HomPPI have been implemented in a server available at: http://homppi.cs.iastate.edu/.

## Methods

### Datasets

Five datasets were used in this paper:

• Nr6505 - For analyzing the protein interface conservation.

• Oblig94 and Trans135 - For comparing the degree of conservation of protein interfaces in transient/obligate binding proteins.

• Benchmark180 - For evaluating the prediction performance of HomPPI.

• S1 and S2 - For evaluating the performance of NPS-HomPPI on interfaces of disordered proteins.

• nr_pdbaa_s2c - For BLASTP searching for close sequence homologs

#### Nr6505

We extracted a maximal non-redundant set of known protein-protein interacting chains from the Protein Data Bank (PDB) [71] available on 2/4/2010. We used the following steps to build Nr6505 to eliminate the influence of over-represented protein families in PDB:

1. Extract all the X-ray derived protein structures with resolution 3.5 Å or better in PDB. Remove proteins with less than 40 residues. We obtained 102,853 protein chains.

2. Remove redundancy of the resulting dataset in step 1 using PISCES[103]. All the remaining sequences have less than or equal to 30% sequence similarity. We obtained 6505 chains.

#### Oblig94 and Trans135

This dataset of 94 obligate protein-protein dimer complexes and the dataset of 135 transient dimer complexes was obtained from a large non-redundant dataset of 115 obligate complexes and 212 transient complexes (3.25 Å or better resolution, determined using X-ray crystallography) previously generated by Mintseris and Weng [76] to study the conservation of protein-protein interfaces. In ordered to exclude the influence of other types of interfaces, we extracted 94 obligate dimers and 135 transient dimers from the original dataset and get Oblig94 and Trans135. In Oblig94, 1QLA has been superseded by 2BS2. In Trans135, 1DN1 and 1IIS have been superseded by 3C98 and 1T83, respectively, and 1F83, 1DF9, 4CPA and 1JCH have since been deemed as obsolete and hence discarded from PDB.

#### Benchmark180

We tested NPS-HomPPI on a benchmark dataset manually collected and used as evaluation dataset by Bradford and Westhead [79]. This dataset consists of 180 protein chains taken from 149 complexes; 36 of these are involved in enzyme-inhibitor interactions, 27 in hetero-obligate interactions, 87 in homo-obligate interactions, and 30 in non-enzyme-inhibitor transient (NEIT) interactions.

#### Disordered protein datasets S1 and S2

We evaluated the performance of NPS-HomPPI on a non-redundant disordered dataset that has been recently collected by Meszaros et al [87]. S1 consists of 46 complexes of short disordered and long globular proteins. S2 consists of 28 complexes of long disordered and long globular proteins. Note that a protein complex e.g., 1fv1 C:AB formed by a disordered protein C with two ordered proteins A and B, yields two sets of interface residues for C (corresponding to interfaces between C with A and C with B). As a result, 46 complexes in S1 and 28 complexes in S2 (respectively) correspond to 56 and 40 interfaces of disordered proteins. We focused on cases in which NPS-HomPPI is able to identify Safe/Twilight/Dark zone homologs for the query proteins resulting in NPS-HomPPI interface predictions for 28 out of 56 and 31 out of 40 interfaces of disordered proteins in S1 and S2 respectively.

#### BLAST nr_pdbaa_s2c

This dataset is used for BLASTP searches. We used the fasta files from S2C database [104] to generate our BLAST database nr_pdbaa_s2c. We removed proteins with resolution worse than 3.5 Å from S2C fasta formatted database. We built a non-redundant database for BLAST queries from the S2C fasta formatted database. To generate the non-redundant BLAST database, we grouped proteins with identical sequences into one entry. We used the resulting database to search for homologs of a query sequence using BLASTP 2.2.22+ [77]. There are 36,352 sequences and 9,549,671 total residues in nb_pdbaa_s2c.

### Interface Definition

This paper adopts a stringent definition of protein-protein interfaces. Surface residues are defined as residues that have the relative solvent accessible area (RASA) at least 5% [84]. Interface residues are defined as surface residues with at least one atom that is within a distance of 4 Å from any of the atoms of residues in the chain. The ratios of interface residues versus the total number of residues for the datasets used in this work are summarized in Table 4. Interface information was extracted from the ProtInDB server http://protInDB.cs.iastate.edu.

**Table 4:** The Proportion of Interface Residues in Datasets used in this Study.

Dataset	Number of Interface Residues^a^	Total Number of Residues^b^	% Interface Residues
**Nr6505**	145,498	1,377,630	10.6%

**Benchmark180**	6,401	43,013	14.9%

**Trans135**	6,460	55,217	11.7%

**Oblig94**	10,273	55,400	18.5%

**Disordered S1^c^**	585	1,171	50.0%

**Disordered S2^c^**	1,797	11,400	15.8%

^a ^When a chain interacts with more than one other chain, the interfaces are counted separately. For example, for protein complex 2phe C:AB, the interface of C with A and the interface of C with B are regarded as two disordered interfaces.

^b ^Residues that missing from PDB structures are not counted.

^c ^For disordered interface datasets S1 and S2, only disordered interfaces are counted.

### Mapping Interfaces in Structures to Sequences

We label the protein sequences as interface or non-interface residues (according to the definition of interface residues given above) as follows: We first calculate the relevant distances between atoms using the atom coordinates in ATOM section in PDB files. Then, by associating the ATOM section to residues in the SEQRES section, we can map the corresponding residues to protein sequences. However, various errors in PDB files make this a non-trivial task. Hence, we used the mapping files from S2C database, which offers corrected mapping information from ATOM section to residues in the SEQRES section of PDB files, to map interfaces determined in structures to full sequences.

### NCBI BLAST Parameters

The amino acid substitution matrix and gap cost are essential parameters that need to be specified in BLAST searches. In this study, we used the substitution matrices and gap costs recommended for the different query lengths [105] (See Table 5).

**Table 5:** BLAST Substitution Matrices and Gap Costs used for BLASTP searches in this paper.

Query Length	Substitution Matrix	Gap Costs
<35	PAM-30	(9,1)

35-50	PAM-70	(10,1)

50-85	BLOSUM-80	(10,1)

85	BLOSUM-62	(10,1)

### Performance Evaluation

To evaluate the extent to which protein interfaces are conserved in query-homolog pairs and to estimate the performance of HomPPI and other predictors that we compare with in predicting the interface residues of a novel protein (i.e., one not used to train the predictor), we consider several standard performance measures including sensitivity (recall), specificity (precision), accuracy and Matthews correlation coefficient (CC) [106]. Specifically, for each test protein *i*, we calculate the corresponding performance measures for each protein *i *as follows:

where *TP_i_*, *FP_i_*, *TN_i _*and *FN_i _*are respectively the number of interface residues of protein *i *that are correctly predicted to be interface residues, the number of residues of protein *i *that are incorrectly predicted to be interface residues, the number of residues of protein *i *that are correctly predicted to be non-interface residues, and the number of residues of protein *i *that are incorrectly predicted to be non-interface residues.

We calculate the *protein-based *overall performance measures as follows:

where *N *is the total number of test proteins.

These measures describe different aspects of predictor performance. The overall sensitivity is the probability, on average, of correctly predicting the interface residues of a given protein. The overall specificity is the probability, on average, that a predicted interface residue in any given protein is in fact an interface residue. The overall accuracy corresponds to the fraction of residues in any given protein, on average, that are correctly predicted. The overall Matthews correlation coefficient measures of how predictions correlate, on average, with true interfaces and non-interfaces.

Often it is possible to trade off one performance measure (e.g., specificity) against another (e.g., sensitivity) by varying the threshold that is applied to the prediction score to generate the binary (interface versus non-interface) predictions. Hence, we include of the overall sensitivity against overall specificity for different choices of the threshold. The resulting specificity-sensitivity plots or precision-recall plots show the trade-off between sensitivity and specificity and hence provide a much more complete picture of predictive performance.

The performance measures described above provide an estimate of the reliability of the predictor in predicting interface residues of a novel *protein*. It is worth noting that most of the papers in the literature on interface residue prediction report performance measures by averaging over *residues *(as opposed to proteins). The *residue-based *overall performance measures are calculated as follows:

Residue-based specificity-sensitivity plots in this case show how the trade-off between specificity^R ^and specificity^R ^is obtained by varying the threshold applied to the prediction score. The residue-based performance measures provide an estimate of the reliability of the predictor in correctly labelling a given *residue*. However, in practice, it is useful to know how well a predictor can be expected to perform on a given protein sequence as opposed to a residue. sensitivity^P^, specificity^P^, accuracy^R^, and CC^P ^are more informative than their residue-based counterparts. Hence, in this paper, we report results based on the protein-based measures although, for the purpose of comparison with other published methods, we include the results based on the residue-based measures in Supplementary Materials in HomPPI website.

### Interface Conservation (*IC*) Scores

In protein interface conservation analysis, we used the CC (defined above) as a measure of the extent to which the interface residues in query protein are similar to those in a putative homolog. For clarity, we refer this measure as the Interface Conservation (*IC*) score.

### NPS-HomPPI

NPS-HomPPI is a Non-Partner-Specific Homologous Sequence-Based Protein-Protein Interface Prediction algorithm. NPS-HomPPI is based on the conclusion from statistical analysis of protein interface conservation on Nr6505, Trans135 and Oblig94, i.e., that protein interfaces are conserved across close sequence homologs.

**Figure 11:**
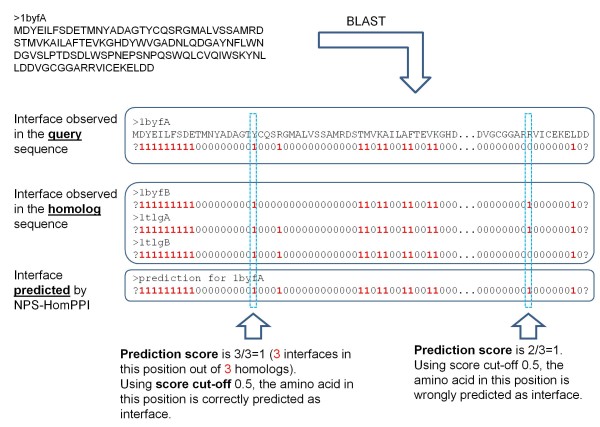
**An example of Interface Residue Prediction using NPS-HomPPI**. The sequence of the query protein 1 byf chain A is BLASTed against nr_pdb_s2c database. In this case, 3 sequences meet the thresholds set by NPS-HomPPI for "close homolog" in Safe Zone or Twilight Zone defined in Table 1. If there are more than K = 10 homologs met the zone thresholds in Table 1, regression equation 1 is used to determine the nearest K homologs for final prediction. For each position in the alignment, an amino acid residue in the query sequence is predicted to be an interface residue if the majority of the amino acid residues in the alignment are interface residues. Otherwise, it is predicted to be a non-interface residue. Interface residues are denoted by red 1's; Non-interface residues are denoted by black 0's. Question marks denote residues for which coordinates are missing from PDB files.

As illustrated in Figure 11, NPS-HomPPI predicts interface residues in a query protein based on the known interface residues of a selected subset of homologs in a sequence alignment. Homologs of the query protein sequence are identified by searching the nr_pdb_s2c database using BLASTP. Note that, in our experiments, in order to allow unbiased evaluation of the performance of NPS-HomPPI, the query sequence itself and sequences that share a high degree (≥95%) of amino acid sequence identity with, and are from the same species as the query sequence are deleted from the set of putative homologs.

If at least one homolog in the Safe Zone is found by the BLASTP search, NPS-HomPPI uses the Safe Zone homolog(s) to infer the interfaces of the query protein. Otherwise, the search is repeated for homologs in the Twilight and Dark Zones. If NPS-HomPPI cannot find homologs in any of the three zones, it does not provide any predictions. The default zone boundaries used by NPS-HomPPI (and hence the parameters used in NPS-HomPPI search for homologs of a query sequence) is based on our interface conservation analysis on the dataset of transient dimers Trans135 (Table 1). The choice of these default parameter thresholds for NPS-HomPPI is intentionally rather conservative; the thresholds can be relaxed if additional information is available (e.g., if we know that the query protein is an obligate binding protein). The *IC *score of each of the homologs of a query sequence in the alignment returned by BLASTP is predicted using the regression model for the *IC *score (see eq. 1) from the BLASTP statistics for the alignment of each homolog with the query sequence. For a given query sequence, at most *K closest *(Safe, Twilight, or Dark Zone homologs, as the case may be, in that order) are selected from the alignment of the query sequence with its homologs to be used to infer the interface residues of the query sequence. In our experiments, *K*, the maximum number of homologs used in the prediction was set equal to 10. At most *K *homologs of the query sequence are determined by ranking the homologs in the alignment in decreasing order of their predicted *IC *scores and choosing (at most) *K *Safe zone homologs (or Twilight zone homologs if no Safe zone homologs exist or Dark zone homologs if neither Safe nor Twilight zone homologs exist). Once the (at most) *K *closest homologs to be used for predicting the interface residues of the query sequence are chosen, each residue in the query sequence is labelled as an interface or non-interface residue based on the majority (over the set of at most *K *closest homologs of the query sequence) of the labels associated with the corresponding position in the alignment. More specifically, each of the at most *K *homologs provides a positive vote for a given position in the query sequence if the corresponding residue of the homolog is an interface residue; and a negative vote if it is a non-interface residue. The prediction score of NPS-HomPPI for that position in the query sequence is simply the number of positive votes divided by the total number of votes. A query sequence residue with a HomPPI score ≥0.5 is predicted to be an interface residue (See Figure 11 for an example); otherwise, it is predicted to be a non-interface residue. This procedure can be seen as an application of the (at most) *K *nearest neighbor classifier at each residue of the query sequence.

#### NPS-Interface Conservation As a Function of Sequence Alignment

We built a linear model for NPS-interface conservation based on the most important sequence alignment statistics identified in the PCA analysis: *logEVal*, *Positive Score*, *logLAL*.

The model is(1)

Variables, parameter estimates and coefficients are shown in Table 6. All the coefficients are significant.

**Table 6:** Variables, Parameter Estimates and Significance Values for the Linear Model for NPS-Interface Conservation.

Variable	Parameter Estimate	Standard Error	t Value	Pr > |t|
*β_0_*	-0.5655	0.0080	-70.66	<.0001
*β_1_*	-0.0004	0.0000	-23.30	<.0001
*β_2_*	0.0037	0.0000	54.44	<.0001
*β_3_*	0.1057	0.0011	94.62	<.0001

### PS-HomPPI

PS-HomPPI predicts the interface residues in a protein chain based on the known interface residues of its closest homo-interologs. Given a query protein A and its interaction partner B, PS-HomPPI first identifies the set homo-interologs of A-B using BLASTP to identify the homologs of A and homologs of B. From the BLASTP results, we identify a set of homo-interologs that meet sequence similarity thresholds (determined based on the results of our partner-specific interface conservation analysis, as described in the ***Results ***Section). We discard the whole PDB complex that contains A-B, to ensure an objective assessment of the reliability of our prediction procedure. For query A-B and its homologous interacting pair A'-B', we also discard the interacting protein pair A'-B' if A and A' or B and B' share ≥95% sequence identity and belong to the same species.

PS-HomPPI uses homo-interologs in the Safe and Twilight Zones to make predictions. The zone boundaries were determined using Trans135 and are shown in Table 7. The PS-HomPPI prediction process is similar to that of NPS-HomPPI in that it progressively searches for homointerologs from higher, then lower, homology zones: i.e., if PS-HomPPI cannot find at least one homo-interolog in the Safe Zone, it next looks for homo-interologs in the Twilight Zone.

**Table 7:** Boundaries of Safe, Twilight and Dark Zones used by PS-HomPPI.

	*LogEVal*	≤ -100
Safe Zone	*PositiveS*	≥70%
	*Frace_AA_*_'_	≥80%
	*Frace_BB_*_'_	≥80%
	*LogEVal*	≤ -50
**Twilight Zone 1**	*PositiveS*	≥60%
	*Frace_AA_*_'_	≥60%
	*Frace_BB_*_'_	≥60%

	*LogEVal*	≤ 1
**Twilight Zone 2**	*PositiveS*	≥55%
	*Frace_AA_*_'_	≥40%
	*Frace_BB_*_'_	≥40%

	*LogEVal*	≤ 1
**Dark Zone**	*PositiveS*	≥0
	*Frace_AA_*_'_	≥0
	*Frace_BB_*_'_	≥0

PS-HomPPI predicts whether an amino acid in query sequence A is an interface residue or not based on the corresponding position in its alignment with (at most) *K *of the closest homo-interologs of A-B (based on their predicted *IC *scores). In our experiments, *K *was set equal to 10. Given a query-partner pair A-B, we label each position in the amino acid sequence of protein A as an interface or non-interface based on whether or not a majority of the corresponding positions of the homologs of A within the homo-interologs of A-B are interface residues. More specifically, each of the at most *K *homo-interologs provides a positive vote for a given position in the query protein sequence A if the corresponding residue of its homolog A' in its homo-interolog is an interface residue; and a negative vote if it is a non-interface residue. The prediction score of PS-HomPPI for that position in the query sequence is simply the number of positive votes divided by the total number of votes. A residue in the query protein A with a prediction score ≥0.5, is predicted as interface, otherwise, it is predicted as non-interface.

#### PS-Interface Conservation As a Function of Sequence Alignment

We built a linear model for PS-interface conservation based on the important sequence alignment statistics identified in the PCA analysis: *logEVal*, *Positive Score*, *Frac_AA_*_' _and *Frac_BB_*_'_, where

A-B is query protein pair and A'-B' is the homo-interolog of A-B. *EVal_AA_*_' _and *EVal_BB_*_' _are the *EVal *between A and A', and between B and B'. *positiveS_AA_*_' _and *positiveS_BB_*_' _are the BLAST *Positive Score *between A and A', between B and B'. The model is(2)

Variables, parameter estimates and coefficients are shown in Table 8. All the coefficients are significant.

**Table 8:** Variables, Parameter Estimates and Significance Values for the Linear Model for PS-Interface Conservation.

Coefficient	Parameter Estimate	Standard Error	t Value	Pr > |t|
*β*_0_	-0.505	0.040	-12.62	<.0001
*β*_1_	0.001	0.000	6.16	<.0001
*β*_2_	0.009	0.001	14.6	<.0001
*β*_3_	0.341	0.027	12.54	<.0001
*β*_4_	0.205	0.028	7.4	<.0001

## Availability and Requirements

* Project name: HomPPI

* Project home page: http://homppi.cs.iastate.edu/

* Programming language: Perl

## Authors' contributions

All authors of this research paper have directly participated in the design, implementation, or analysis of this study.
